# Mineral Composition and Quality Traits of *Morchella* spp. Harvested from Forest Plantations and Native Forests in South-Central Chile

**DOI:** 10.3390/jof12080588

**Published:** 2026-08-08

**Authors:** Tatiana Escobar-Hernández, Rodrigo Segura, Claudia Muñoz-Espinoza, Mauricio Sanz-Rocha, Erick Zagal, Valeria Velasco, Mhartyn Elso, Yudith Guillén, Macarena Gerding, Daniel Chávez, Ángela Machuca

**Affiliations:** 1 Programa de Magíster en Ciencias Agronómicas, Facultad de Agronomía, Universidad de Concepción, Campus Chillán, Av. Vicente Méndez 595, Chillán 3801061, Chile; tf.escobarhz@gmail.com; 2 Departamento de Ciencias y Tecnología Vegetal, Universidad de Concepción, Campus Los Ángeles, Juan Antonio Coloma 0201, Los Ángeles 4451032, Chile; msanz2126@gmail.com (M.S.-R.); mhelso@udec.cl (M.E.); yguillen@udec.cl (Y.G.); danielchavez@udec.cl (D.C.); 3 Departamento de Química de los Materiales, Facultad de Química y Biología, Universidad de Santiago de Chile, Avenida Libertador Bernardo O’Higgins 3363, Santiago 9170022, Chile; rodrigo.segura@usach.cl; 4 Departamento de Producción Vegetal, Facultad de Agronomía, Universidad de Concepción, Campus Chillán, Av. Vicente Méndez 595, Chillán 3801061, Chile; claudiaalexmunoz@udec.cl (C.M.-E.); mgerding@udec.cl (M.G.); 5 Departamento de Suelos y Recursos Naturales, Facultad de Agronomía, Universidad de Concepción, Campus Chillán, Av. Vicente Méndez 595, Chillán 3801061, Chile; ezagal@udec.cl; 6 Departamento de Producción Animal, Facultad de Agronomía, Universidad de Concepción, Campus Chillán, Av. Vicente Méndez 595, Chillán 3801061, Chile; vvelasco@udec.cl

**Keywords:** wild edible mushroom, *Morchella andinensis*, accumulation factor, mineral composition, nutritional quality, bioactivity

## Abstract

Morels are highly valued edible mushrooms due to their ecological and nutraceutical importance. However, nutritional studies in South America are still scarce. This study evaluated the mineral, nutritional, and bioactive properties of *Morchella eximia*, *M. importuna*, *M. tridentina*, and the endemic *M. andinensis*, which naturally fruits in forest plantations (FPs) and native forests (NFs) in south-central Chile. Soils differed significantly, indicating varied fertility levels across morel habitats. Ascocarps from all species exhibited significant accumulation of K and P, with moderate accumulation of Ni and Zn. Cd and Pb remained within internationally accepted safe limits, while As was undetectable. The determination of the translocation factor, performed only on *M. andinensis* and *M. tridentina*, indicated a preferential translocation of K and P to the pileus. Differences were observed among the species in terms of phenolic content and antioxidant activity. A proximate analysis revealed that Chilean morels had elevated levels of protein and fiber, similar to those reported in morels from other latitudes. An exploratory multivariate analysis suggested that the observed variation in mineral composition and bioactive and nutritional properties of Chilean morels likely reflects the combined influence of soil characteristics, forest environment, and genotype traits. These preliminary results are the first to describe traits related to the nutritional quality of *Morchella* spp. from south-central Chile.

## 1. Introduction

The genus *Morchella* comprises ascomycete fungi highly valued for their gastronomic appeal [1,2], nutritional composition, and bioactive potential [3,4,5,6]. Commonly known as morels, these edible mushrooms are prized for their distinctive flavor, texture, and rich profile of bioactive compounds. This has made them a valuable fungal resource with potential applications in the food, nutraceutical, and pharmaceutical industries [3,7,8,9,10].

Interest in morels has recently grown due to increasing commercial demand [11,12,13]. However, the origin of the fruiting bodies, or ascocarps, is particularly important, as those of wild origin develop in heterogeneous natural environments and may better reflect the interaction between the fungus and soil conditions [14]. In contrast, the available substrates and nutrients in cultivation systems are largely controlled [10,13,15,16,17]. This distinction is crucial when evaluating the presence of specific potentially toxic metallic elements, as the risk and magnitude of metal bioaccumulation can vary considerably between wild and cultivated morels [18,19,20].

Edible wild mushrooms are known for their ability to absorb minerals from their growing environment [17,21,22], including essential elements such as sodium (Na), potassium (K), calcium (Ca), manganese (Mn), zinc (Zn), copper (Cu), and iron (Fe) [7,17,23], which are necessary for metabolism. Also, they absorb non-essential or potentially toxic elements such as cadmium (Cd), lead (Pb), arsenic (As), and chromium (Cr) [17,19], hazardous for human health [24,25,26]. This ability gives fungi biotechnological potential for the bioremediation of contaminated environments [27,28]. However, it also raises concerns about the nutritional quality of ascocarps intended for human consumption [3,17,29].

Various physiological mechanisms are involved in the metal absorption and accumulation in fungi, including biosorption in cell walls [30,31,32], active incorporation through membrane transporters [16,33], and differential translocation between the stipe and pileus of the ascocarps [7,34]. The determination of parameters such as the accumulation factor (AF) and translocation factor (TF) allows one to assess the efficiency with which fungi absorb elements from the soil and redistribute them internally [17,18,35]. This provides insight into their nutrition and detoxification strategies.

Furthermore, the presence and accumulation of metals have been recorded in *Morchella* species collected in mountainous regions and in soils with anthropogenic influences or particular characteristics [14,24,29,36]. However, most of these studies have focused on the Northern Hemisphere and often lack precise species identification, making comparisons between results difficult. In the Southern Hemisphere, particularly in Chile, morels are mainly harvested from the wild because artificial cultivation has not yet been developed commercially [37,38]. Large quantities of morels are harvested in areas with high levels of anthropogenic disturbance, as well as in more preserved forests with native vegetation. Most of the harvest is exported, while a small amount is allocated for local consumption [39]. However, information about the quality of the harvested product is still scarce, and there is a lack of data integrating the nutritional composition and presence of metals in the ascocarps of formally identified *Morchella* species.

In this context, the aim of this study is to evaluate some quality attributes of the ascocarps of the *Morchella* spp. collected from native forests and forest plantations in south-central Chile, which exhibit varying degrees of anthropogenic disturbance. In addition, this study investigates the possible influence of environmental conditions at collection sites on the quality attributes of Chilean morels. These results should provide the preliminary scientific data necessary for a comprehensive assessment of this valuable edible resource, as well as help identify any potential risks associated with its consumption. Furthermore, the results should provide a foundation for the sustainable and safe management of this resource by promoting best practices for harvesting and habitat conservation in south-central Chile.

## 2. Materials and Methods

### 2.1. Study Sites

**Figure 1:**
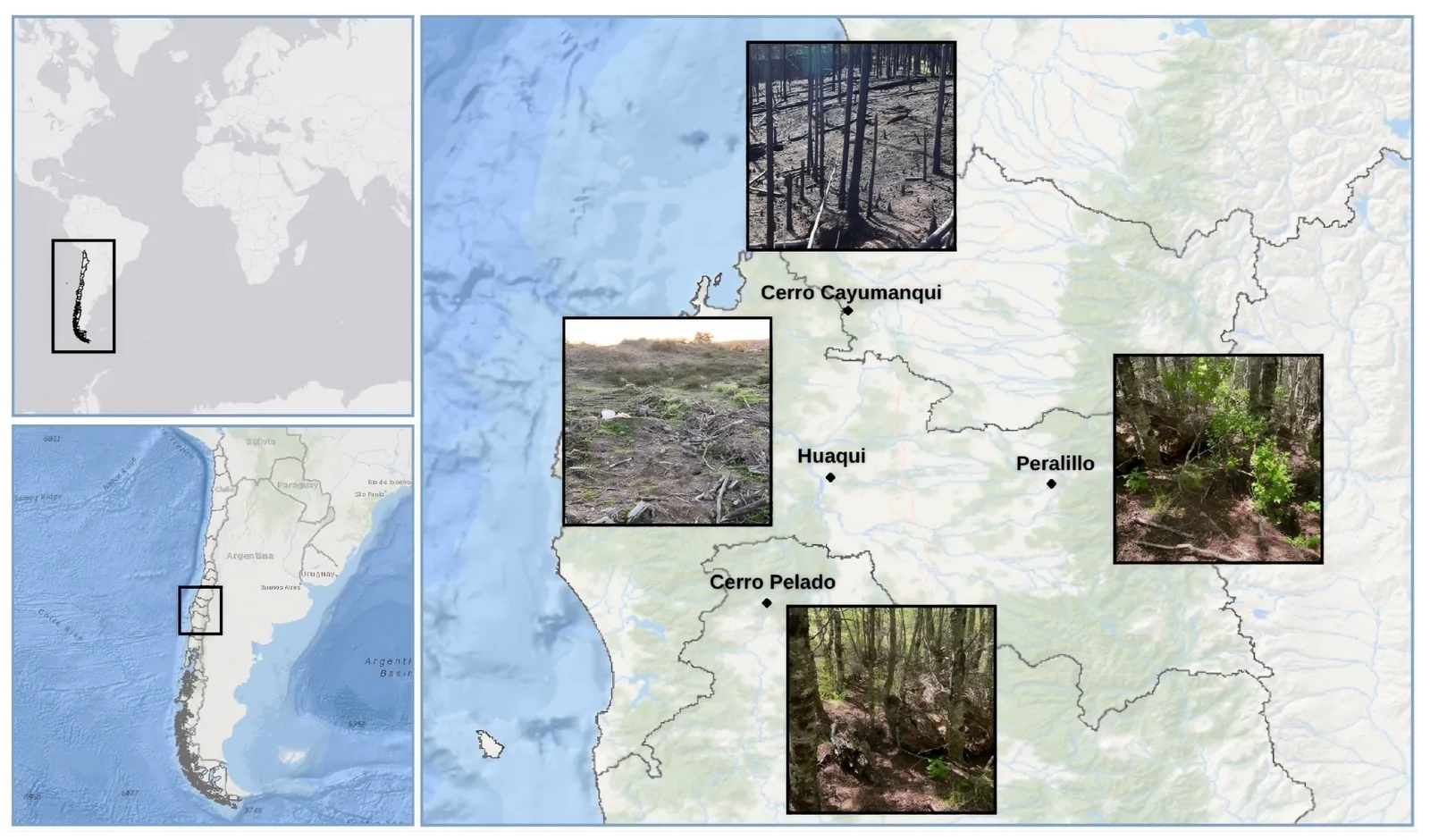
Geographical location and visual characteristics of the four *Morchella* spp. natural fruiting sites in south-central Chile correspond to forest plantations disturbed by fire (Cerro Cayumanqui) or logging (Huaqui), and native forests (Peralillo and Cerro Pelado). The images provide overviews of the environmental conditions at each study site.

Four sites were selected in south-central Chile (latitude 36.7–37.8 °S), with a temperate Mediterranean climate, where the natural fruiting of the morel has been reported. These sites represent different types of vegetation cover and disturbance conditions. Two sites are relict native forests, dominated by species of the genus *Nothofagus*: Peralillo (Pe) and Cerro Pelado (CPe) sites. Two other sites correspond to *Pinus radiata* forest plantations, which have been harvested or affected recently by fires: Huaqui (Hu) and Cerro Cayumanqui (CCa) sites, respectively. Thus, the study sites exhibit a variety of disturbances, and physical characteristics allow for the wide distribution of *Morchella* spp. (Figure 1; Supplementary Table S1).

### 2.2. Sampling

#### 2.2.1. Collection of Ascocarps and Soils

The morel ascocarps were harvested between August and November during the 2023 and 2024 seasons. At each study site, 3 m^2^ plots were demarcated. Fresh ascocarps were collected from these plots, individually packaged in paper bags, and transported to the laboratory at 4 °C, where they were carefully cleaned of any soil or leaf litter.

For the soil samples, 50 g of soil was extracted from the base of each specimen. Three independent soil samples, corresponding to three different specimens, were collected at each site. In addition, within each plot, composite soil samples were collected from the surface layer (0–20 cm) in an “X” pattern. Each sample consisted of five 500 g subsamples. All soil samples were packaged in plastic bags and transported to the laboratory under refrigerated conditions (4 °C).

#### 2.2.2. Sample Preparation

From each site, approximately 250 g of fresh morels were collected, and a composite sample (200 g) was obtained for proximate analysis (Section 2.8). Additionally, individual samples (*n* = 3) were reserved for mineral content analysis (Section 2.4), phenol content analysis, antioxidant activity analysis (Section 2.7), and species molecular identification. To prepare the ascocarps, the samples were dehydrated and freeze-dried. Then, they were ground to a particle size of 1 mm and stored at room temperature in hermetically sealed containers with silica gel. The soil samples were homogenized and dried in an oven at 105 °C for 24 h. Then, the composite samples were sieved to a size of 2 mm and stored at 4 °C until processing. For mineral determination, the samples were sieved to 0.63 µm and stored in hermetically sealed containers with silica gel until analysis.

#### 2.2.3. Species Identification

All morel specimens collected in the field were identified based on micro- and macroscopic morphological descriptions of the fresh ascocarps [38,40], and representative ascocarps from each morphological group were analyzed using molecular techniques. Approximately 100 mg of dry pileus tissue was suspended in 250 µL of TL buffer E.Z.N.A. Tissue DNA Kit (Omega Bio-tek, Inc. Norcross, GA, USA), following the manufacturer’s instructions. The ITS region (ITS1–5.8S–ITS2) was amplified by PCR using the ITS1 and ITS4 universal primers [41], according to the conditions described by Machuca et al. [38]. The PCR products were sequenced by AUSTRAL-Omics (Valdivia, Chile). The sequences obtained were edited, aligned, and compared with reference sequences available in GenBank using the Geneious v2025.0.1 software. A maximum-likelihood phylogenetic tree was constructed in MEGA v5.2 software [42], using the GTR+I+G model, and the robustness of the tree was evaluated with 1000 bootstrap replicates. In addition, a BLAST (https://blast.ncbi.nlm.nih.gov/Blast.cgi) search was performed to confirm the identity and phylogenetic similarity of the sequences obtained. A voucher specimen of each identified species was deposited in the fungarium of the Fungal Biotechnology Laboratory at the University of Concepción, under the UDEC-LAF code.

### 2.3. Physical and Chemical Analysis of Soils

The soils were characterized by determining their pH, organic matter (OM) content, carbon/nitrogen (C/N) ratio, and cation exchange capacity (CEC). The sand, silt, and clay fractions were also quantified to establish the textural class. Chemical analyses were performed according to the methods described by Sadzawka et al. [43] and Sadzawka [44], while physical analyses were performed according to the procedures reported by Sandoval et al. [45].

### 2.4. Analysis of Essential and Non-Essential Mineral Elements

For the analysis of mineral elements, the ascocarps and soil samples were mineralized, for which sieved samples (0.63 µm) of 0.2 g of ascocarps and dry soil were weighed. Then, acid digestion was performed in Teflon bombs, adding 8 mL of 65% HNO_3_ and 2 mL of 30% H_2_O_2_, using a microwave digestion system (Milestone Ethos Easy, Sorisole, Italy) for 90 min at 200 °C. After cooling, the samples were filtered through 0.45 µm filters and then gauged to 25 mL with ultrapure water (Milli-Q, Darmstadt, Germany). Ca, Cu, Fe, K, Na, P, and Zn were determined in the mineralized samples using flame atomic absorption spectrometry (FAAS) with an Analytik Jena AnovA 350 (Jena, Germany)(Cu, Fe, and Zn) and on a Thermo Scientific ICE 3000 Series instrument (Cambridge, United Kingdom)(Ca, K, Na, and P). The calibration curves were made using standards at four different concentrations, manually adjusted for each element based on the linear response range of the device. Analytical quality was verified in all cases by evaluating the coefficient of determination (R^2^ > 0.95). Meanwhile, As, Cd, Co, Cr, Mn, Ni, and Pb were determined using a Thermo Scientific ICE 3500 Series (Cambridge, United Kingdom) graphite furnace atomic absorption spectrometer (GF-AAS) under the manufacturer’s optimal conditions. For these analyses, an external standard of 50 g L^−1^ was used for each element, and calibration curves were made, and the linearity was confirmed using the coefficient of determination (R^2^ > 0.95). Three categories were established to analyze the mineral elements found in soils and ascocarps: (i) major essential elements: Ca, K, Na, and P (g kg^−1^); (ii) essential trace elements: Co, Cr, Cu, Fe, Mn, Ni, and Zn (mg kg^−1^); and (iii) trace elements potentially harmful to health: As, Cd, and Pb (mg kg^−1^).

### 2.5. Accumulation Factor (AF)

The accumulation factor of mineral elements by *Morchella* spp. was calculated as the ratio of the element concentration in the ascocarps to its concentration in the corresponding soil, using the following Formula (1) [46]:

(1) $$AF=\frac{Concentration\;of\;the\;element\;in\;the\;ascocarps\;({mg\;kg}^{-1})}{Concentration\;of\;the\;element\;in\;the\;soil\;({mg\;kg}^{-1})}$$

AF values > 1 indicate that the ascocarps are actively accumulating the element from the soil, while AF < 1 suggests that the ascocarps are excluding or absorbing it in smaller proportions [47].

### 2.6. Translocation Factor (TF)

To determine the translocation factor (TF), samples of the pileus and stipe of morel ascocarps collected in native forests were analyzed separately. Subsequently, the TF of mineral elements was calculated as the ratio of the pileus concentration to the stipe concentration using the following Formula (2) [48]:

(2) $$TF=\frac{Concentration\;of\;the\;element\;in\;pileus\;({mg\;kg}^{-1})}{Concentration\;of\;the\;element\;in\;the\;stipe\;({mg\;kg}^{-1})}$$

TF values > 1 indicate that the ascocarp actively translocates the element from the stipe to the pileus, whereas TF < 1 indicates a reduced element mobilization [24].

### 2.7. Analysis of Total Phenols and Antioxidant Activity

#### 2.7.1. Total Phenols

Dried and ground samples of the ascocarps were weighed to 0.2000 g into 50 mL Falcon tubes and then 10 mL of 70% acetone was added. The suspension was placed in an ultrasonic bath (Biobase UC-40A, Jinan, China) for 30 min, after which it was centrifuged at 4400 rpm for 30 min at 4 °C. The supernatant was recovered and stored in an ice bath for later total phenol analysis. The Folin–Ciocalteu reagent (Merck, Germany) was used to quantify total phenols in the extracts according to Slinkard and Singleton [49]. The results were expressed as tannic acid equivalents per gram of dry material (mg TAE g^−1^ DM).

#### 2.7.2. Antioxidant Activity

The antioxidant activity was determined using a methanolic extract of the dried and ground ascocarps. Then, 7.5 mg of the sample was weighed and processed according to the methodology of Hatano et al. [50]. The mixture was kept in an ultrasonic bath for 24 h at 4 °C in darkness. Afterward, it was centrifuged at 4400 rpm for 30 min at 4 °C, and the supernatant was recovered and stored in ice bath until analysis. Antioxidant activity was determined using the stable 2,2-diphenyl-1-picrylhydrazyl (DPPH) free radical, following the methodology of Gursoy et al. [19]. The results were expressed in terms of percentage inhibition of the DPPH free radical (I%), calculated according to the following Formula (3):

(3) $$I\%=\frac{A\;control-A\;sample}{A\;control}\times 100$$

### 2.8. Proximate Composition

To determine the proximate composition, composite samples (200 g of fresh material) of ascocarps were collected from native forest sites (Cerro Pelado and Peralillo) and forest plantation sites (Huaqui and Cerro Cayumanqui). The proximate analysis was performed separately for each site on composite samples of ascocarps that had been previously identified (Section 2.2.3). These procedures followed the official methodologies of the Association of Official Analytical Chemists [51]. Moisture content (MC) was determined by drying the samples in an oven at 105 °C until they reached a constant weight. The MC was then calculated using the following Formula (4):

(4) $$MC\left(\%\right)=\frac{Weight\;fresh\;-\;Weight\;dry}{Weight\;fresh}\times 100$$

The following parameters were quantified: crude protein (CP) using Kjeldahl (method 991.20), crude fiber (CF) by digestion (method 926.09), total ash (TA) by muffle furnace calcination (method 942.05), and (EE) by Soxhlet extraction (method 920.39C). The non-nitrogen extract (NNE) content was calculated by difference according to the following Formula (5):

(5) $$NNE(\%)=100-(CP\left(\%\right)+EE\left(\%\right)+CF\left(\%\right)+TA\left(\%\right))$$

The metabolizable energy (E) was calculated using Atwater’s Equation [52] (6):

(6) $$E\left({kcal\;100\;g}^{-1}\right)=4\times CP\left(\%\right)+9\times EE\left(\%\right)+4\times NFE\left(\%\right)$$

### 2.9. Statistical Analysis

Whit the exception of the soil characterization data and proximate composition data, all the analyses were performed in triplicate, and results were expressed as the mean ± standard deviation. Statistical analysis was performed using the RStudio (version 2026.07.1) [53], with a significance level of *p* < 0.05. Assumptions of normality and homogeneity of variances were verified for each data set. An analysis of variance (ANOVA) was performed on variables that accomplished these assumptions, followed by an LSD (least-significant-difference) multiple-comparison test. When statistical assumptions were not met, transformations and nonparametric tests were applied. The Cr values used to determine the TF were transformed using log(x + 1) to meet the assumptions required for the ANOVA. Variables that did not reach normality, such as the Cu and Ni content in ascocarps and the As, Cd, Co, Cu, Ni, Na, and Pb content for determining AF and As for determining TF, were analyzed using the nonparametric Kruskal–Wallis test, followed by Dunn’s multiple-comparison test. An exploratory principal component analysis (PCA) was also performed with 21 variables related to ascocarp quality using the R statistical software and the FactoMineR (version 2.11) library. Then, to identify the most contributing variables, a correlation analysis was performed between the variables and the first two components derived from PCA. The analysis selected the variables that were significantly correlated (*p* < 0.05) [54]. The soil characterization and proximate composition data were obtained from one composite sample per study site and are therefore presented descriptively, without inferential statistical analysis.

## 3. Results and Discussion

Although morels are commercially cultivated in countries such as China [55], wild harvesting remains the primary method of acquiring and consuming them in many other countries. In Chile, *Morchella* spp. can be found in pristine native forest ecosystems such as those in Patagonia [38,56]. However, it can also be found outside of Patagonia in forest plantations (FPs) and native forests (NFs) that have experienced varying degrees of anthropogenic disturbance [40]. Most of the morels harvested in Chile are mainly exported to European markets. Only a small portion is sold for consumption in local markets [39]. Due to the economic importance of this export resource, this study evaluated some quality attributes of *Morchella* spp. ascocarps harvested from different forest environments in south-central Chile. In addition, this study provides an initial exploration of the possible influence of environmental conditions at collection sites on the quality of morels.

### 3.1. Characteristics of Study Sites

The study sites where *Morchella* spp. fruited differed in their degree of disturbance (Supplementary Table S1). The FP sites showed the most altered environments. Cerro Cayumanqui (CCa) was affected by an extended fire in February 2023, and Huaqui (Hu) was subjected to logging for timber harvest in December 2022. In contrast, the NF sites, Peralillo (Pe) and Cerro Pelado (CPe), represent relicts that, despite conserving native vegetation, are surrounded and have been partially invaded by exotic plantations, mainly in the case of the Pe site. The soils at these study sites (NF and FP) also showed contrasting physical and chemical properties (Table 1).

The pH of the soil was similar and slightly acidic at all sites, mainly at site CPe (5.90) in NF. The carbon–nitrogen ratio (C/N) was also similar at all sites, and particularly low values (0.10) were observed at the CCa site in FP. In contrast, organic matter (OM) content and cation exchange capacity (CEC) parameters showed the greatest differences between sites. The OM content varied widely, ranging from over 10% at the NF sites to 2.8% at the FP sites. In the case of CEC, the highest value was observed at the Pe site in NF (20.91 cmol^+^ kg^−1^) and the lowest at the Hu site in FP (3.26 cmol^+^ kg^−1^). These parameters (OM and CEC) are indicators of superior fertility and greater nutrient retention, associated with the accumulation of leaf litter and a lower degree of disturbance [57,58,59]. This is consistent with observations at NF sites. In contrast, sites of harvested or burned forest plantation (FP) had low OM content and CEC, suggesting lower soil quality and reduced nutritional support capacity [60,61].

Differences in soil texture were observed among the sites related to varying proportions of sand, silt, and clay. The FP sites were notable for having soils with higher sand (Hu) and clay (CCa) content. These results indicated that the study sites have contrasting soil conditions, ranging from lighter soils with low water retention (Hu) to finer soils with greater retention capacity (CCa). These results showed that morels can fruit under a wide range of soil conditions, demonstrating remarkable ecological plasticity in response to different soil qualities and textures.

### 3.2. Morchella Species

This study used morphological descriptions and sequencing of the ITS region [38,40] to confirm the identification of four *Morchella* species from the Elata clade in south-central Chile (Supplementary Table S2; Figures S1 and S2). Each species was associated with a particular site, and there was no overlap in their distribution. At the FP sites, all the specimens analyzed from the fire-affected site (CCa) showed 99.7–100% similarity to *M. eximia*. This is consistent with the strictly pyrophilic nature of *M. eximia* described in the literature, which requires fire events to trigger the production of fruiting bodies [1,56,62]. Conversely, the specimens from the logged forest plantation (Hu) showed 100% similarity to *M. importuna*, which suggests an affinity of this species for highly disturbed environments. Depending on their location, the NF specimens exhibited 100% similarity with *M. tridentina* (Pe) and between 99.84 and 100% similarity with *M. andinensis* (CPe) (Supplementary Table S2). These results are consistent with previous studies describing the presence of these *Morchella* species in less disturbed environments dominated by *Nothofagus* spp. [38,56,63]. Although knowledge of species diversity is of key scientific significance, it should be noted that morels are currently marketed and exported as a single product without differentiation by species or harvesting environment.

### 3.3. Soil Mineral Content

The soils at the sites where *Morchella* spp. fruited in south-central Chile varied significantly in the concentration of major, essential, and potentially toxic elements (Table 2). In most cases, the levels of analyzed mineral elements were significantly higher in soils at FP sites than at NF sites. This difference reflects the influence of anthropogenic disturbance and the edaphic origin of the study sites [14,17,64].

As for the major essential elements (g kg^−1^), the highest levels of calcium (Ca), potassium (K), and sodium (Na) were found in the soils at the FP sites. The highest level of K was found in the burned FP site, while the highest levels of Ca and Na were found in the logged FP site. High variability was observed for Na, with the maximum value found at the burned FP site. Such patterns are consistent with the effects of forest disturbances, particularly wildfires. Studies have demonstrated that these disturbances can enhance nutrient and mineral redistribution in the soil, sometimes very irregularly through the ash [64,65]. On the other hand, the NF sites had the lowest concentrations of Ca, K, and Na, but the highest levels of phosphorus (P). This was especially remarkable at the Pe site, where the levels were significantly higher than at the other sites (Table 2).

Contrasting patterns were recorded regarding the trace elements (mg kg^−1^) in the soils of the PF and NF sites. The highest contents of cobalt (Co) and iron (Fe) were detected at the burned FP site and Pe site (NF). These values were significantly higher than those at the other sites. The highest content for chromium (Cr) was detected at the burned FP site, followed by the Pe site (NF). Meanwhile, the highest content of nickel (Ni) was detected at the logged FP site. The manganese (Mn) content was high and similar across sites, whereas the zinc (Zn) and copper (Cu) content was low and similar as well.

In terms of potentially toxic trace elements the levels of arsenic (As) and lead (Pb) were significantly higher in the soils at the burned FP site, followed by the NF sites. Meanwhile, the highest cadmium (Cd) content was detected at the NF site (Pe) (Table 2).

Harvested FP had the highest Ca and Ni contents, which may be linked to the removal of vegetation cover and exposure of the mineral horizon. Consistently with the literature, burned FP had high concentrations of K, Cr, As, and Pb, indicating that fires can mobilize nutrients and metals immediately, albeit temporarily [64,65,66,67]. Overall, these results suggest that the type of disturbance, in combination with the intrinsic physicochemical properties of the soil, may contribute to the observed variation in nutrient and metal contents [17], which could affect morel fruiting at study sites. Nevertheless, these patterns should not be attributed exclusively to environmental conditions, as genotype physiological traits of morels could also influence the response of this edible mushroom.

### 3.4. Mineral Content of Morchella *spp.*

As a quality trait of the ascocarps of *Morchella* spp. collected from the various study sites, the mineral composition was assessed in terms of essential and potentially toxic non-essential elements. The ascocarps collected at the FP and NF sites showed significant variations in the concentration of essential and potentially toxic mineral elements. For most elements, the highest concentrations were detected in the ascocarps of species harvested from the FP site that were burned (*M. eximia*) or logged (*M. importuna*) (Table 3).

The general trend in the concentration of major elements in morel ascocarps was K > P >> Ca ≈ Na. Among the species, *M. importuna* and *M. andinensis* showed the highest Ca content, whereas K, the most abundant macronutrient in all species, was significantly higher in *M. andinensis*. In addition, *M. importuna* also had the highest Na concentration, followed by *M. eximia*, which together with *M. andinensis* had the highest P concentrations (Table 3).

Trace elements showed the general trend Ni > Mn > Cr >> Fe > Co > Zn > Cu in the morel ascocarps (Table 3). Among the FP species, *M. eximia* exhibited the highest concentrations of Cu and Ni, while *M. importuna* had the highest concentrations of Co and Fe. Both species had significantly higher Cr content in their ascocarps than the other species. The NF species *M. andinensis* showed the highest concentration of Mn in its ascocarps, followed by *M. eximia*, though the difference was not significant. Zn was the only trace element that showed no significant differences among the different *Morchella* species.

Regarding potentially toxic elements, the Cd concentration was significantly higher in *M. eximia*, while the Pb concentration was higher in *M. importuna*; both species were found in FP. However, the Pb concentration was also high in *M. andinensis* from NF (Table 3). Under the analytical conditions of this study, none of the *Morchella* species had detectable concentrations of As in their ascocarps. This is despite the fact that significant quantities of As and Pb were found in the soils of the study sites, at much higher concentrations than Cd, mainly at the burned FP site. However, As was not detected in the ascocarps of any of the *Morchella* species under our analysis conditions. This absence is particularly relevant from a nutritional point of view, considering that As is one of the most toxic and highest-risk metalloids in wild fungi, having been detected in other edible species such as *Amanita caesarea* or *Boletus edulis* [29,36,68].

**Figure 2:**
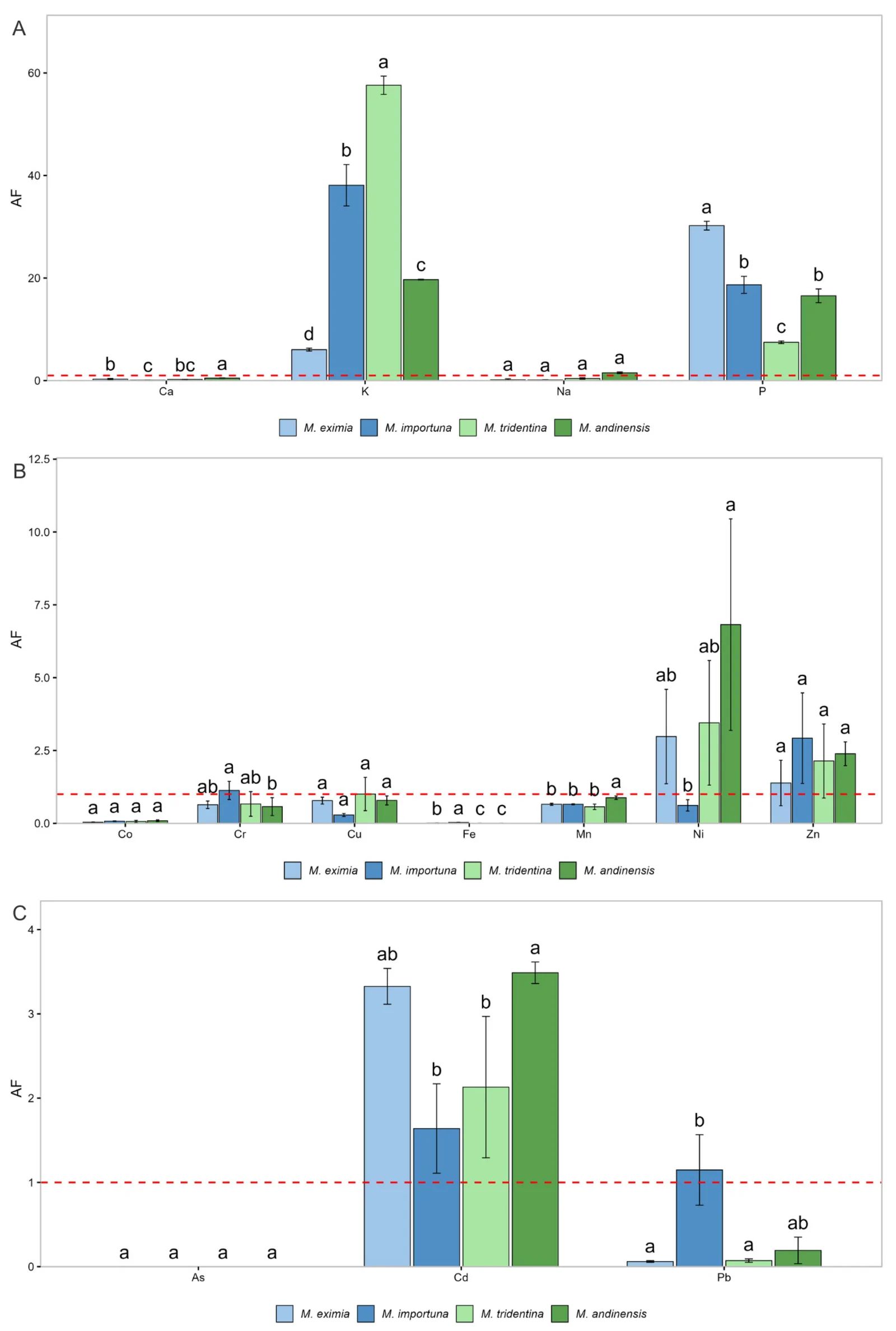
Accumulation factor (AF) of (**A**) major essential elements, (**B**) essential trace elements, and (**C**) potentially harmful trace elements in morel ascocarps collected in forest plantations (*M. eximia* and *M. importuna*) and native forests (*M. tridentina* and *M. andinensis*) in south-central Chile. Values correspond to means ± standard deviation (*n* = 3). Different letters above the bars indicate significant differences between species (*p* < 0.05). The dashed red line indicates the AF threshold = 1, signifying the accumulation of the element in the ascocarp relative to that in the soil.

The determination of mineral element accumulation in the morel ascocarps using the accumulation factor (AF) revealed significant differences between species, depending on the element type (Figure 2). All *Morchella* species from FP and NF showed a high accumulation of K and P (AF >> 1), while Ca and Na accumulation was limited (AF < 1) (Figure 2A). In the case of K, the marked accumulation (AF 6–58) showed the general trend *M. tridentina* > *M. importuna* > *M. andinensis* > *M. eximia*. Despite having lower AF values than K, P was also present in large quantities (AF 7.5–30) in the ascocarps of the different species, following the trend *M. eximia* > *M. importuna* > *M. andinensis* > *M. tridentina* (Figure 2A). The results suggest that morels may function as superaccumulators of macronutrients with a notably species-specific accumulation capacity. Thus, although the contents of these elements (K and P) were not the highest in the soil, the intrinsic capacity of the species enabled them to accumulate these elements in their ascocarps. This behavior is consistent with previously reported studies in other edible fungi such as *B. edulis* and *Cantharellus cibarius*, where K and P also accumulated significantly, which would be related to their physiological relevance [20,68,69]. On the other hand, the accumulation of these elements highlights their nutritional importance. Both elements play key roles in the human body [69,70], such as electrolyte balance, muscle function, and blood pressure regulation in the case of K, and bone and tooth formation in the case of P [71,72,73]. In contrast, the low accumulation of Ca and Na (AF < 1) in the ascocarps of all species suggests the presence of exclusion mechanisms or low ionic mobility, consistent with the osmotic and homeostatic regulatory strategies described for ectomycorrhizal fungi [18,20]. The low Ca content in morels agrees with reports on most edible fungi, where this element is generally found in low levels due to its limited mobility and poor intracellular accumulation capacity [19,22,74]. Although low Ca content reduces its contribution to the diet, this characteristic is considered physiologically normal within the group of edible fungi [68,69]. Nevertheless, a low concentration of Na is a positive attribute from a nutritional perspective since it is desirable in human nutrition and contributes to regulating blood pressure and water balance [23,25,70,75]. These results confirm that, although morels are not a significant source of Ca, their low Na content and high K and P content reinforce their value as a healthy, functional food.

A significant accumulation (AF > 1) of Ni and Zn was observed among the essential trace elements. The high Ni accumulation (AF 3–6.8) following the trend *M. andinensis* > *M. tridentina* > *M. eximia*. *M. importuna* was the only species that showed limited Ni accumulation (AF 0.62) in its ascocarps. The AF values for Zn were lower than those observed for Ni; however, Zn accumulated in the ascocarps of all the species (AF 1.4–2.9) (Figure 2B). Although Ni plays an important physiological role at low concentrations, excess Ni can be toxic to the human body [76,77]. However, the concentrations detected in *Morchella* spp. remain within safe consumption ranges [24,26,78], so moderate levels could contribute to essential micronutrient intake without posing a health risk. The other trace elements exhibited a limited (Cr, Cu, and Mn) to very limited (Co and Fe) accumulation in the ascocarps of the different *Morchella* species (Figure 2B). This is similar to reports regarding other wild edible mushrooms [68,79,80]. In the case of Fe, one of the most abundant elements in the soils at the study sites, the lowest accumulation values (AF 0.05) were observed, suggesting an active exclusion or very limited absorption. This could be related to the low bioavailability of Fe in its ferric state [69,81].

Regarding potentially harmful elements, the AF values indicated the accumulation of Cd and Pb (AF > 1) (Figure 2C). The Cd exhibited a significant accumulation in all species (AF 1.6–3.5), in the order *M. andinensis* ≈ *M. eximia* > *M. tridentina* > *M. importuna*. For Pb, only *M. importuna* showed accumulation of this element (AF 1.15), while the other species had limited accumulation in their ascocarps (≤0.19). These results are consistent with those reported for other species in the genus, such as *M. esculenta*, *M. galilaea*, and *M. conica*, in which a greater accumulation of Cd than for Pb has also been observed [24,26,74,82]. Cd accumulation appears to be a common trait in the genus *Morchella*, possibly due to its ability to synthesize chelating compounds such as chitin, melanins, and polysaccharides, which immobilize metals and reduce their intracellular toxicity [18,83]. Several authors have suggested that low concentrations of Cd could even play an ecological role in wild fungi by acting as a defense mechanism against insect or microorganism predation, a phenomenon also observed in plants [68,84]. The Cd concentrations recorded in *Morchella* spp. did not exceed the permissible limits established by the FAO/WHO [78] for human consumption, maintaining levels within safe margins and ensuring its safety. Thus, the accumulation patterns of essential and non-essential elements in morel ascocarps suggest a strategy involving a significant accumulation of essential elements (K, P, Zn, Ni), selective exclusion of others (Ca, Na, Fe), and limited retention of potentially toxic elements. These patterns suggest physiological regulatory mechanism balancing nutrition, detoxification, and environmental responses, which could be influenced by morel genotype and habitat-specific conditions [24]. Nevertheless, to support the suggested interpretations, a larger number of study sites in south-central Chile that represent different environmental conditions, as well as a greater number of ascocarps collected per study site, are needed.

**Figure 3:**
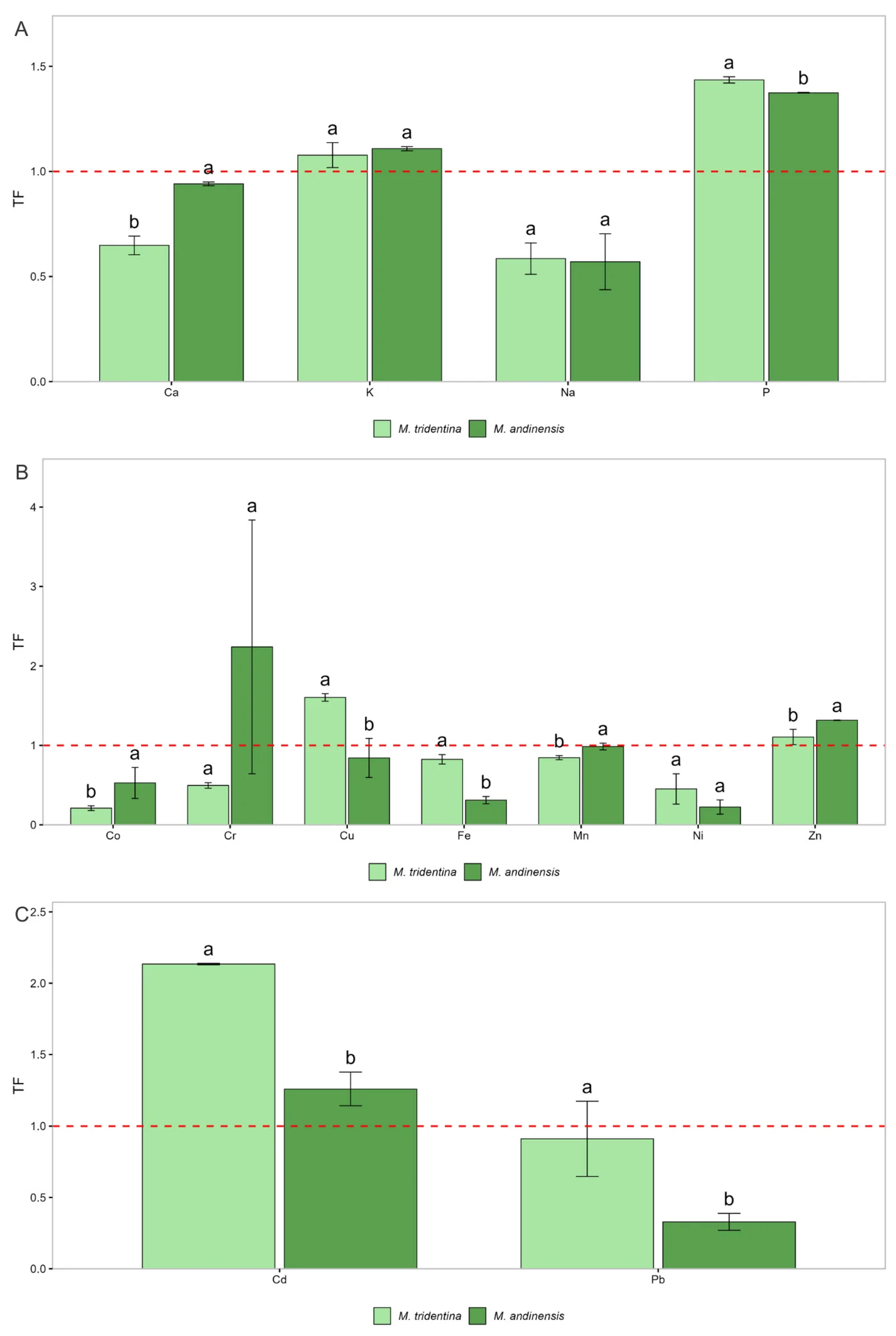
Translocation factors (TFs) of (**A**) major essential elements, (**B**) essential trace elements, and (**C**) potentially harmful trace elements between pileus and stipe in *M. tridentina* and *M. andinensis* from native forest. Values correspond to means ± standard deviation (*n* = 3). Different letters above the bars indicate significant differences between species (*p* < 0.05). The red dashed line indicates the TF threshold = 1, representing an equal distribution of the element between the structures, with values > 1 indicating greater translocation to the pileus.

The translocation factor (TF) of elements was only assessed in species from NF, given that *M. andinensis* is a species endemic to South America [38,63], making it a particularly relevant fungal resource of ecological and local interest. *M. tridentina* was also included in this analysis despite being a transcontinental species to facilitate a direct comparison with *M. andinensis*, as both species fruit in the same type of NF ecosystem. The translocation of mineral elements from the stipe to the pileus differed between the two species depending on the element evaluated (Figure 3). Among the major essential elements (Figure 3A), only K and P showed active translocation to the pileus (TF > 1) in both species, with slightly higher P values (1.44) in *M. tridentina*. In the case of Ca, *M. andinensis* exhibited an almost equal distribution between the two structures, with a TF value of 0.94, which was significantly higher than the value of 0.65 obtained for *M. tridentina*. In contrast, TF values < 1 were obtained for Na in both species (0.57–0.59), indicating a preferential accumulation in the stipe. Thus, while Ca and Na exhibited limited mobility and remained in the stipe (TF < 1), K and P actively moved toward the pileus in both species (TF > 1). This is consistent with the role of K and P in respiration and reproduction processes [20,55], and the lower mobility of Ca and Na in fungal tissues [68,69].

Regarding the essential trace elements, only Cr, Cu, and Zn showed translocation to the pileus (TF > 1), with significant differences among species (Figure 3B). These differences could reflect physiological variations in transport efficiency and the metabolic utilization of metals [68,82,83]. For Cr, a trend toward transport to the pileus was only detected in *M. andinensis* (TF 2.24), and for Cu, this translocation occurred only in *M. tridentina* (TF 1.60). Both species showed Zn translocation of the element toward the pileus (TF > 1), with the highest values for *M. andinensis*. TF values < 1 were observed for Co, Fe, and Ni in both species, and for Mn, the values were close to one (TF ≈ 1), mainly in *M. andinensis*. Of the potentially toxic elements, only Cd showed preferential accumulation in the pileus (TF > 1), mainly in *M. tridentina* (TF 2.13) (Figure 3C). In contrast, greater retention of Pb was detected in the stipe of both species (TF < 1).

Since the pileus is the most consumed part of the morel ascocarps, the fact that there is a higher accumulation of essential elements in these structures is relevant from a nutritional perspective. However, the presence of potentially toxic elements could pose a safety risk [24,84]. Consequently, the combined evaluation of AF and TF provides complementary insights into mineral accumulation and translocation in *Morchella* spp. However, the evaluation of TF was limited to *M. andinensis* and *M. tridentina*. Therefore, to better understand metal translocation in morels from south-central Chile, research should expand to include *M. eximia* and *M. importuna*, facilitating a comparison between morels from NF and FP.

In Chile, there are no reference values for Cd or Pb in wild or cultivated edible mushrooms. Therefore, it is not possible to establish specific consumption recommendations. However, the levels detected in Chilean morels are within internationally accepted safe ranges [69,78,84], meaning only high and frequent consumption would pose a risk. This emphasizes the importance of regulations and systematic monitoring to ensure the safety of wild fungal products.

### 3.5. Bioactivity of Morchella *spp.*

Other quality attributes measured in the morel ascocarps included biological activity, which was determined by the phenolic compound content and antioxidant activity, and nutritional quality, which was determined by proximate composition.

**Figure 4:**
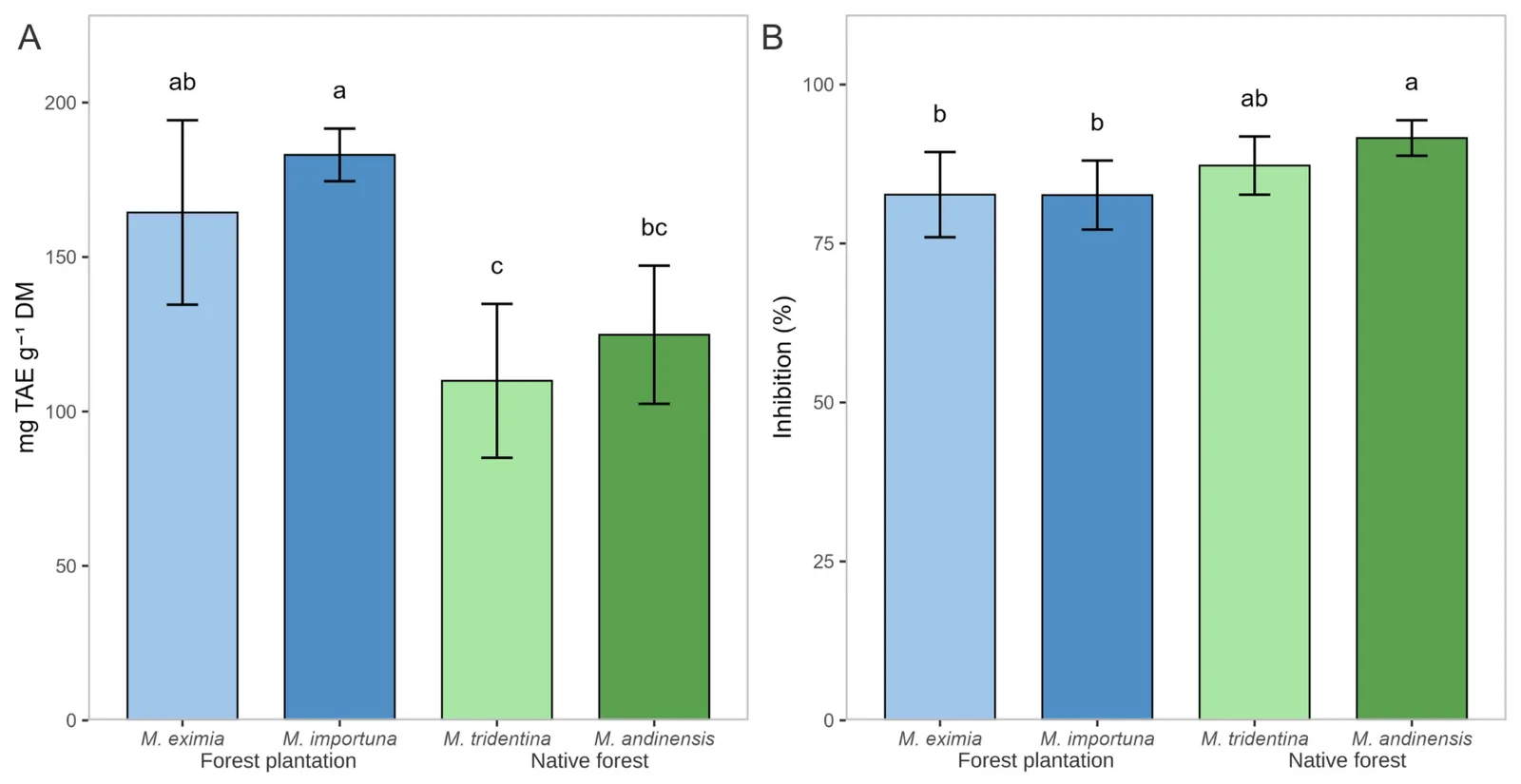
Total phenol content (mg TAE g^−1^ DM) (**A**) and the antioxidant activity (**B**) expressed as inhibition (%) of the DPPH radical by the morel ascocarps collected from forest plantations (*M. eximia* and *M. importuna*) and native forests (*M. tridentina* and *M. andinensis*) in south-central Chile. Values correspond to the mean ± standard deviation (*n* = 3). Different letters above the bars indicate significant differences between species (*p* < 0.05).

The total phenol content in the ascocarps showed significant differences among species from different habitat types (Figure 4A). The two species associated with FP, *M. eximia* and *M. importuna*, exhibited the highest total phenol values (164.4 and 183.1 mg TAE g^−1^ DM, respectively), with no significant differences between them. However, both NF species, *M. tridentina* and *M. andinensis*, presented similar levels of total phenols (109.9 and 124.8 mg TAE g^−1^ DM, respectively), lower than those of the FP species, although *M. andinensis* did not differ significantly from *M. eximia*. The antioxidant activity of ascocarps (Figure 4B), determined as DPPH radical inhibition (%), showed significant differences among species from different habitats. The ascocarps of the FP species, *M. eximia* and *M. importuna*, showed similar values of DPPH radical inhibition, with averages close to 80%, with no statistical differences between them. *M. andinensis* showed antioxidant activity (close to 90%) significantly higher than *M. importuna* and *M. eximia* from FP. Meanwhile, *M. tridentina* showed an intermediate value, statistically comparable to the other species.

The observed differences in the bioactive potential of *Morchella* spp. suggest that this variation could reflect the combined influence of factors, including the species genotype and the environmental conditions at the collection sites where the ascocarps developed [2,4,85]. The ascocarps collected in FP exhibited a higher total phenolic content than those collected in NF. This could be related to the more stressful environmental conditions in FP, such as lower plant diversity [86], and exposure to a highly disturbed environment, factors that stimulate the synthesis of secondary metabolites with protective functions in fungi [2,19,87,88]. In contrast, NF ascocarps, particularly those of *M. andinensis*, showed high antioxidant activity with low phenolic content, suggesting the presence of antioxidants other than phenolic compounds in these morels or the presence of compounds with greater DPPH radical inhibitory capacity [2,4,19,85]. It is widely recognized that non-phenolic compounds, including vitamins (C and E), certain amino acids, terpenoids, and melanin, among others, have the ability to reduce the free radical DPPH [89]. The results obtained align with reports on other wild edible mushrooms, where antioxidant activity does not always correlate with phenolic content [90,91], but rather with the presence of polysaccharides or bioactive peptides [5,7,85,92,93].

### 3.6. Proximate Composition of Morchella *spp.*

The proximate composition was determined from one composite sample of ascocarps from a single *Morchella* species collected at each of the four study sites (Table 4). Due to the limited number of ascocarps available at certain study sites and the significant sample volume required for proximate determination, it was not feasible to obtain biological replicates. Consequently, the data are presented descriptively, without statistical analysis (Table 4). Overall, all morel samples exhibited high crude protein contents (31.08–36.61% DM) and crude fiber contents (15.27–18.50% DM) [4,7]. Ether extract remained low in all samples (3.80–6.06% DM) [3,70], whereas total ash ranged from 7.91 to 10.46% DM. Nitrogen-free extract varied between 31.48 and 39.25% DM, resulting in total carbohydrate contents ranging from 48.85 to 55.64% DM. The estimated energy value ranged from 308.34 to 321.80 kcal 100 g^−1^ (Table 4). These preliminary results suggest that wild *Morchella* species from south-central Chile are a nutritionally valuable food source characterized by high protein and dietary fiber contents and low fat levels, reinforcing their potential as functional foods and sources of bioactive compounds [69,70,94,95].

Although proximate composition was determined from one composite sample collected at each study site, the nutritional values obtained were comparable to those reported for other wild *Morchella* species from the Northern Hemisphere, including *M. sextelata* and *M. esculenta* [2,3,4]. In Chile, previous studies have reported the nutritional composition of wild morels from Patagonia [96]. The results indicate high levels of protein and ash, with low levels of fat, but without species-level discrimination reported. Likewise, recent studies have shown that cultivated morels exhibit nutritional profiles similar to those of wild specimens [3,12], suggesting that the nutritional characteristics of *Morchella* are generally conserved across species and growing conditions.

Variation in proximate composition of Chilean morels may reflect the combined influence of species identity, the physiological stage of the ascocarps at harvest, and local environmental and edaphic conditions, all of which have previously been recognized as key factors influencing the nutritional composition of edible mushrooms [18,20]. However, to verify these observations, it is necessary to expand the study by increasing the number of ascocarps collected for each species (biological replicates) and the collection sites in south-central Chile.

The present study provides the first species-level characterization of the preliminary proximate composition of *Morchella* species from south-central Chile. Although the limited sampling design does not allow the independent effects of species identity, study site, and environmental conditions to be distinguished, the results provide valuable baseline information for future studies.

### 3.7. Principal Component Analysis (PCA)

**Figure 5:**
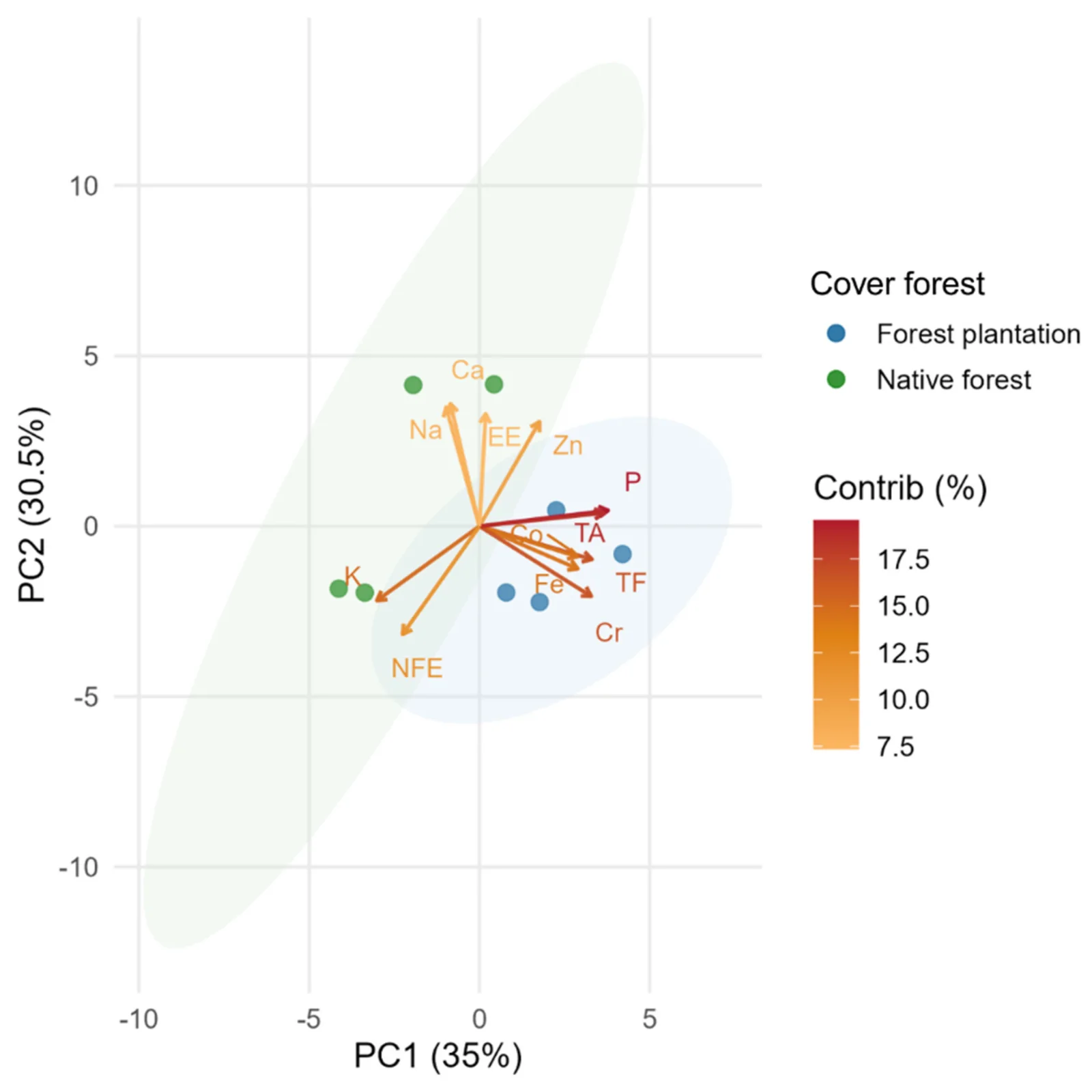
Principal component analysis (PCA) of the quality traits of morel ascocarps collected from FP (blue) and NF (green). The points represent the study sites, and the ellipses indicate the 95% confidence interval for each forest cover. Arrows represent the variables included in the analysis, and their color intensity indicates their percentage contribution to the first two principal components (PC1 = 35% and PC2 = 30.5%).

A principal component analysis (PCA) was performed to explore the relationships between the quality traits of morel ascocarps and their possible association with environmental conditions at collection sites. This multivariate approach allows visualization of the clustering patterns among ascocarps from native forests (NFs) and forest plantations (FPs). It also facilitate the preliminary identification of the variables that may contribute most to their differentiation (Figure 5).

The exploratory PCA explained 65.5% of the total variance (PC1 = 35%, PC2 = 30.5%), revealing consistent differences between ascocarps collected in FP and NF. The variables with the highest significant correlation (*p* ≤ 0.05) with the principal axes were P, total ash content (TA), Ca, Na, total phenols (TP), ethereal extract (EE), non-nitrogenous extract (NNE), Cr, Zn, K, Fe, and Co. PC1 was positively associated with P, TA, TP, Cr, Fe, and Co, and negatively associated with K. This indicates that these variables primarily contributed to differentiating ascocarps from FP, which have higher concentrations of these substances. By contrast, PC2 was associated with Ca, Na, Zn, and EE, describing a pattern more representative of NF. The analysis shows that ascocarps collected in FP exhibit a more concentrated P and TA profile, while those from NF have higher Ca, Na, and Zn contents.

The association of FP species with elements such as P, Cr, and Fe, as well as higher levels of phenolic compounds, suggests an effect of environmental disturbance on their metabolism. This is consistent with what has been described for other species of the genus under similar conditions [2,85]. Conversely, the relationship between NF species and K and moisture suggests a more balanced chemical profile that is favorable for organoleptic and nutritional quality [3,4]. The preliminary results of the multivariate analysis suggest that the observed variation in the mineral composition, bioactive compound content, and nutritional properties of morels from south-central Chile likely reflects the combined influence of soil characteristics, forest environments, and species-specific characteristics.

## 4. Conclusions

The results of this study provide a preliminary characterization of the mineral composition, nutritional properties, and bioactive compounds in wild *Morchella* species from south-central Chile. These species include the endemic *M. andinensis* and the transcontinental *M. eximia, M. importuna*, and *M. tridentina*. Overall, the results suggest that the observed variation in quality attributes among the studied ascocarps likely reflects the combined influence of genotype characteristics and the edaphic and forest environmental conditions under which Chilean morels developed. Soils in forest plantation (FP) were less fertile and more disturbed, while soils in native forest (NF) were characterized by higher organic matter (OM) and cation exchange capacity (CEC). Differences in soil characteristics were associated with variations in the mineral and bioactive composition of the morel ascocarps. However, given the limited number of sites and morel collections included in the present study, future research should incorporate a larger number of sampling sites, including additional pristine native forests, as well as increased ascocarp collections. This will allow for a more comprehensive evaluation of the relative contribution of genotype traits and edaphic and environmental factors on the quality of Chilean morels.

Mineral analyses revealed that *Morchella* spp. efficiently accumulates essential macronutrients, particularly K in *M. tridentina* and P in *M. eximia*, while controlling the absorption of Ca and Na and selectively accumulating micronutrients such as Ni and Zn. Similarly, the ascocarps showed limited retention of potentially toxic elements, with no detection of As and safe levels of Cd and Pb. This indicates the safety of Chilean morels for human consumption, at least for those harvested at the study sites described here.

Differences in phenolic compound content and antioxidant activity reflect the bioactive potential of species influenced by the environment, with higher phenolic content in FP ascocarps and higher antioxidant capacity in NF ascocarps. The analyzed Chilean morels exhibited a high protein and crude fiber content, with values comparable to those reported for cultivated *Morchella* species in the North Hemisphere. These findings are consistent with the nutritional and functional potential previously described for this edible mushroom.

To the best of our knowledge, this study is the first to describe the mineral composition and quality traits of four species of *Morchella*, including the endemic *M. andinensis*, that fruit in different forest ecosystems in south-central Chile. The study of morel mushrooms yields critical knowledge for the conservation of their natural habitats and the development of sustainable collection strategies that ensure the protection of this valuable Chilean wild export resource.

## Figures and Tables

**Table 1:** Physical and chemical characteristics of soils at the different study sites where *Morchella* spp. fruits naturally in south-central Chile.

Site	pH	OM (%)	C/N	CEC (cmol^+^ kg^−1^)	Sand (%)	Silt (%)	Clay (%)	Textural Class
Burned forest plantation (CCa)	6.50	2.87	0.10	10.91	42.70	26.80	30.50	Clay loam
Logged forest plantation (Hu)	6.40	2.83	0.30	3.26	82.00	13.30	4.70	Loamy sand
Native forest (Pe)	6.20	18.62	0.22	20.91	52.30	27.70	22.00	Sandy clay loam
Native forest (CPe)	5.90	11.37	0.54	8.11	62.40	21.50	16.10	Sandy loam

The values correspond to composite samples (*n* = 5) from each study site. CCa: Cerro Cayumanqui, Hu: Huaqui, Pe: Peralillo, CPe: Cerro Pelado.

**Table 2:** Element contents in soils at the different study sites where *Morchella* spp. fruits in south-central Chile.

Element	Burned Forest Plantation (CCa)	Logged Forest Plantation (Hu)	Native Forest (Pe)	Native Forest (CPe)
Major essential elements (g kg^−1^)				
Ca	1.42 ± 0.09 b	7.58 ± 0.24 a	1.03 ± 0.03 c	1.56 ± 0.01 b
K	5.75 ± 0.13 a	0.78 ± 0.07 c	0.53 ± 0.00 d	2.13 ± 0.02 b
Na	21.42 ± 20.79 a	5.29 ± 0.05 ab	0.57 ± 0.01 b	0.21 ± 0.03 b
P	0.54 ± 0.03 d	0.65 ± 0.02 c	1.71 ± 0.06 a	0.94 ± 0.08 b
Essential trace elements (mg kg^−1^)				
Co	7.33 ± 0.03 a	5.67 ± 0.22 b	7.18 ± 0.03 a	3.88 ± 0.00 c
Cr	22.56 ± 0.73 a	12.40 ± 0.61 c	15.62 ± 0.11 b	7.13 ± 0.52 d
Cu	0.03 ± 0.00 b	0.03 ± 0.00 b	0.05 ± 0.00 a	0.01 ± 0.00 c
Fe	44.84 ± 2.33 a	25.67 ± 2.84 c	46.77 ± 0.71 a	31.95 ± 0.81 b
Mn	21.85 ± 0.55 a	20.99 ± 0.08 ab	20.56 ± 0.74 b	18.62 ± 0.06 c
Ni	7.03 ± 0.54 b	12.03 ± 0.35 a	6.56 ± 0.32 b	1.30 ± 0.01 c
Zn	0.08 ± 0.04 ab	0.02 ± 0.00 c	0.09 ± 0.00 a	0.05 ± 0.00 bc
Trace elements potentially harmful to health (mg kg^−1^)				
As	9.95 ± 1.08 a	1.25 ± 0.29 c	4.38 ± 0.27 b	2.00 ± 0.77 c
Cd	0.34 ± 0.01 b	0.28 ± 0.00 c	0.56 ± 0.05 a	0.27 ± 0.00 c
Pb	8.48 ± 1.46 a	1.60 ± 0.02 d	5.45 ± 0.03 ac	4.03 ± 0.18 c

Values correspond to the mean ± standard deviation (*n* = 3). Different letters in the row indicate significant differences (*p* < 0.05). CCa: Cerro Cayumanqui, Hu: Huaqui, Pe: Peralillo, CPe: Cerro Pelado.

**Table 3:** Element contents in morel ascocarps harvested from forest plantations (*M. eximia* and *M. importuna*) and native forests (*M. tridentina* and *M. andinensis*) in south-central Chile.

Element	*M. eximia*	*M. importuna*	*M. tridentina*	*M. andinensis*
Major essential elements (g kg^−1^)				
Ca	0.38 ± 0.17 b	0.71 ± 0.08 a	0.23 ± 0.02 b	0.76 ± 0.05 a
K	34.72 ± 0.90 b	29.56 ± 0.66 c	30.66 ± 1.14 c	42.03 ± 0.49 a
Na	0.48 ± 0.23 ab	0.64 ± 0.16 a	0.23 ± 0.09 b	0.32 ± 0.01 b
P	16.22 ± 0.57 a	12.05 ± 0.79 b	12.78 ± 0.03 b	15.52 ± 0.13 a
Essential trace elements (mg kg^−1^)				
Co	0.27 ± 0.07 bc	0.42 ± 0.08 a	0.20 ± 0.07 c	0.28 ± 0.05 b
Cr	14.33 ± 2.59 a	13.88 ± 3.19 a	5.63 ± 2.60 b	4.13 ± 2.23 b
Cu	0.03 ± 0.00 a	0.01 ± 0.00 bc	0.01 ± 0.00 ac	0.01 ± 0.00 b
Fe	0.54 ± 0.07 b	0.77 ± 0.05 a	0.24 ± 0.06 c	0.21 ± 0.08 c
Mn	14.28 ± 0.50 ab	13.74 ± 0.37 bc	11.81 ± 2.24 c	16.78 ± 1.48 a
Ni	20.57 ± 9.99 a	7.44 ± 2.41 b	7.62 ± 2.49 b	10.07 ± 4.78 b
Zn	0.09 ± 0.01 a	0.07 ± 0.03 a	0.09 ± 0.04 a	0.09 ± 0.03 a
Trace elements potentially harmful to health (mg kg^−1^)				
As	0.00 ± 0.00 a	0.00 ± 0.00 a	0.00 ± 0.00 a	0.00 ± 0.00 a
Cd	1.13 ± 0.11 a	0.46 ± 0.14 d	0.65 ± 0.13 c	0.90 ± 0.04 b
Pb	0.51 ± 0.00 b	1.83 ± 0.64 a	0.37 ± 0.07 b	0.80 ± 0.63 b

Values correspond to the mean ± standard deviation (*n* = 3). Different letters in the row indicate significant differences (*p* < 0.05).

**Table 4:** Proximate composition of the *Morchella* spp. ascocarps collected from four study sites in south-central Chile.

		Forest Plantation		Native Forest	
		*M. eximia*	*M. importuna*	*M. tridentina*	*M. andinensis*
Moisture content	(%)	83.49	85.49	87.59	91.23
Crude protein	(% DM)	36.61	31.08	32.65	34.79
Crude fiber	(% DM)	15.27	18.50	16.39	18.18
Total ash	(% DM)	10.46	10.38	7.91	9.50
Ethereal extract	(% DM)	4.08	4.77	3.80	6.06
Nitrogen-free extract	(% DM)	33.58	35.27	39.25	31.48
Energy value	(kcal 100 g^−1^)	317.47	308.34	321.80	319.57

Values correspond to one composite ascocarp sample collected at each study site. DM: dry matter.

## Data Availability

The original contributions presented in this study are included in the article. Further inquiries can be directed to the corresponding author.

## Supplementary Materials

The following supporting information can be downloaded at https://www.mdpi.com/article/10.3390/jof12080588/s1. Table S1: Characteristics of the study sites where Morchella spp. fruits naturally in south-central Chile; Table S2: Molecular identification of Morchella spp. collected from different study sites in south-central Chile; Figure S1: Phylogenetic relationships of Morchella spp. from south-central Chile based on ITS sequences; Figure S2: Morel ascocarps from distinct forest environments in south-central Chile.
