# Supplementary material for: Mineral Composition and Quality Traits of Morchella spp. Harvested from Forest Plantations and Native Forests in South-Central Chile
Source: J Fungi (Basel). 2026 Aug 8;12(8):588. doi: 10.3390/jof12080588 (PMC13514789; doi:10.3390/jof12080588)
Supplement: Supplementary file 1 [file jof-12-00588-s001.zip › jof-4435569-supplementary.pdf]

Supplementary Material (Escobar-Hernandez et al.)

Table S1. Characteristics of the study sites where *Morchella* spp. fruits naturally in south-central Chile.

Site	Administrative region	Geographical coordinates	Altitude (m a.s.l)	Predominant vegetation	Type of disturbance
Forest plantation (Cerro Cayumanqui)	Ñuble	36°44'41.11"S 72°34'16.17"O	512	*Pinus radiata* (burned tree debris)	Forest fire
Forest plantation (Huaqui)	Biobío	37°22'9.32"S 72°38'15.43"O	137	*Pinus radiata* (logged tree debris)	Logging
Relic of Native forest (Peralillo)	Biobío	37°23'39.30"S 71°48'32.72"O	621	*Nothofagus* spp.	Relict of native forest with forestry and livestock influence
Relic of Native forest (Cerro Pelado)	La Araucanía	37°50'52.58"S 72°53'6.98"O	955	*Nothofagus* spp.	Human and domestic animal traffic influence

Table S2. Molecular identification of *Morchella* spp. collected from different study sites in south-central Chile.

Site	Specie identified	Closest reference (GenBank access)	BLAST identity (%)
Burned forest plantation (CCa)	*M. eximia*	MT952472.1	100.00
	M. eximia	PV874418.1	99.70
	M. eximia	KM587970.1	99.85
Logged forest plantation (Hu)	*M. importuna*	PV501037.1	100.00
	M. importuna	MH982689.1	100.00
	M. importuna	PV501037.1	100.00
Native forest (Pe)	*M. tridentina*	MN355532.1	100.00
	M. tridentina	KJ439679.1	100.00
	M. tridentina	KJ439679.1	100.00
Native forest (CPe)	*M. andinensis*	MT952467.1	99.84
	M. andinensis	MT952467.1	100.00
	M. andinensis	MT952470.1	100.00

Species identification was based on sequence comparison of the ITS region with reference sequences available in GenBank using BLAST. CCa: Cerro Cayumanqui, Hu: Huaqui, Pe: Peralillo, CPe: Cerro Pelado.

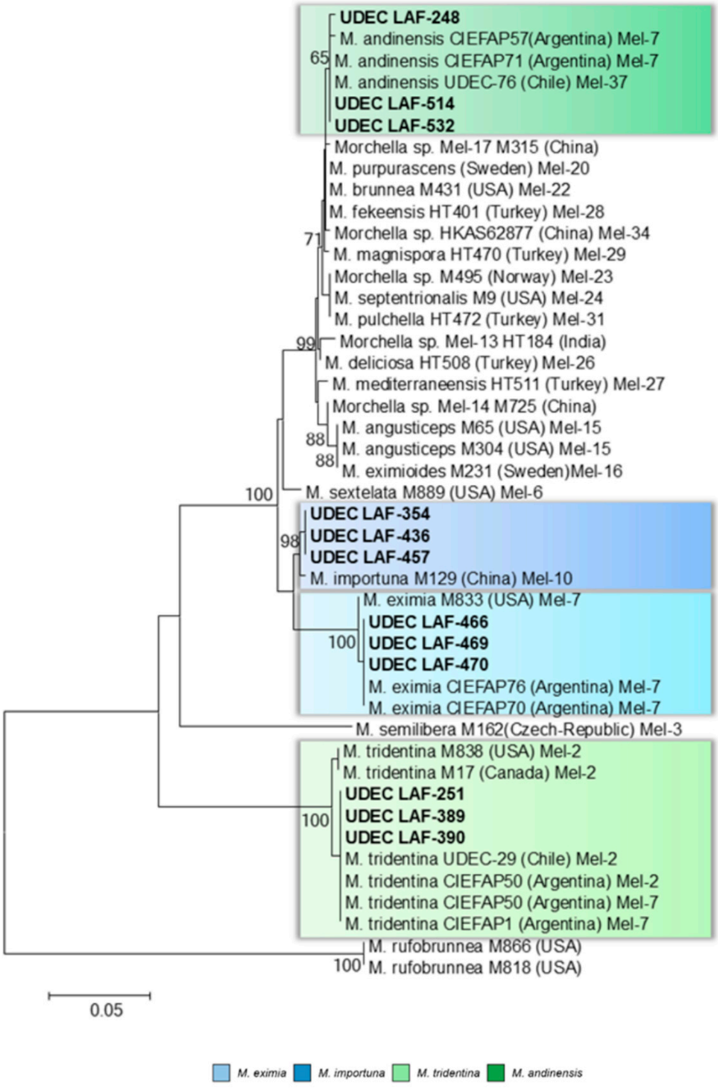

Figure S1. Phylogenetic tree of *Morchella* spp. from south-central Chile, based on ITS region sequences. The analysis included *M. eximia* (Cerro Cayumanqui), *M. importuna* (Huaqui), *M. tridentina* (Peralillo), and *M. andinensis* (Cerro Pelado), along with reference sequences from GenBank. The tree was constructed using the maximum likelihood method with the Kimura 2 + I evolutionary model and 1000 bootstrap replicates. The sequences generated in this study are highlighted in blue tones for strains originating in forest plantations (*M. eximia* and *M. importuna*) and in green tones for those originating in native forests (*M. tridentina* and *M. andinensis*).

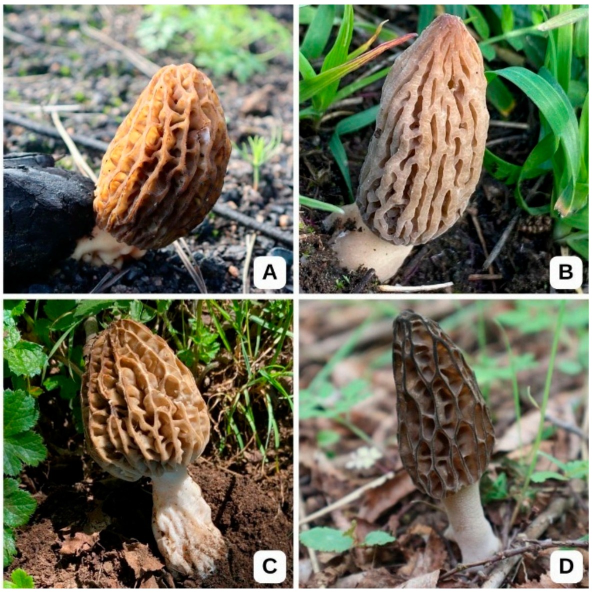

Figure S2. Ascocarps representative of *Morchella* species from different study sites in south-central Chile, identified as *M. eximia* (A), *M. importuna* (B), *M. tridentina* (C), and *M. andinensis* (D). Photographs were taken during the fruiting period in a burned forest plantation (A), a logged forest plantation (B), and a native forest (C and D).
